# Augmented Insulin and Leptin Resistance of High Fat Diet-Fed APPswe/PS1dE9 Transgenic Mice Exacerbate Obesity and Glycemic Dysregulation

**DOI:** 10.3390/ijms19082333

**Published:** 2018-08-08

**Authors:** Yi-Heng Lee, Hao-Chieh Hsu, Pei-Chen Kao, Young-Ji Shiao, Skye Hsin-Hsien Yeh, Feng-Shiun Shie, Shu-Meng Hsu, Chih-Wen Yeh, Hui-Kang Liu, Shi-Bing Yang, Huey-Jen Tsay

**Affiliations:** 1 Institute of Neuroscience, School of Life Science, National Yang-Ming University, Taipei 112, Taiwan; lee.ean91@gmail.com (Y.-H.L.); pattykao60816@hotmail.com (P.-C.K.); k76003@gmail.com (S.-M.H.); flow5168@hotmail.com (C.-W.Y.); 2 Taiwan International Graduate Program in Interdisciplinary Neuroscience, National Yang-Ming University and Academia Sinica, Taipei 112, Taiwan; 3 Institute of Anatomy and Cell Biology, School of Medicine, National Yang-Ming University, Taipei 112, Taiwan; chieh0820@hotmail.com; 4 National Research Institute of Chinese Medicine, Ministry of Health and Welfare, Taipei 112, Taiwan; yshiao@nricm.edu.tw (Y.-J.S.); hk.liu@nricm.edu.tw (H.-K.L.); 5 Institute of Biopharmaceutical Science, National Yang-Ming University, Taipei 112, Taiwan; 6 Brain Research Center, National Yang-Ming University, Taipei 112, Taiwan; skyeyeh@live.com; 7 Aging and Health Research Center, National Yang-Ming University, Taipei 112, Taiwan; 8 Center for Neuropsychiatric Research, National Health Research Institutes, No. 35 Keyan Road, Zhunan Town, Miaoli County 350, Taiwan; fshie@nhri.org.tw; 9 Ph.D. Program for Clinical Drug Discovery from Botanical Herbs, Taipei Medical University, Taipei 110, Taiwan; 10 Institute of Biomedical Sciences, Academia Sinica, Taipei 115, Taiwan

**Keywords:** Alzheimer’s disease, high fat diet, hypothalamus, leptin resistance, insulin resistance

## Abstract

Alzheimer’s disease (AD), a progressive neurodegenerative disease is highly associated with metabolic syndromes. We previously demonstrated that glycemic dysregulation and obesity are augmented in high fat diet (HFD)-treated APPswe/PS1dE9 (APP/PS1) transgenic mice. In the current study, the underlying mechanism mediating exacerbated metabolic stresses in HFD APP/PS1 transgenic mice was further examined. APP/PS1 mice developed insulin resistance and, consequently, impaired glucose homeostasis after 10 weeks on HFD. [^18^F]-2-fluoro-2-deoxy-d-glucose ([^18^F]-FDG) positron emission tomography showed that interscapular brown adipose tissue is vulnerable to HFD and AD-related pathology. Chronic HFD induced hyperphagia, with limited effects on basal metabolic rates in APP/PS1 transgenic mice. Excessive food intake may be caused by impairment of leptin signaling in the hypothalamus because leptin failed to suppress the food intake of HFD APP/PS1 transgenic mice. Leptin-induced pSTAT3 signaling in the arcuate nucleus was attenuated. Dysregulated energy homeostasis including hyperphagia and exacerbated obesity was elicited prior to the presence of the amyloid pathology in the hypothalamus of HFD APP/PS1 transgenic mice; nevertheless, cortical neuroinflammation and the level of serum Aβ and IL-6 were significantly elevated. Our study demonstrates the pivotal role of AD-related pathology in augmenting HFD-induced insulin and leptin resistance and impairing hypothalamic regulation of energy homeostasis.

## 1. Introduction

Alzheimer’s disease (AD) is a progressive neurodegenerative disorder and accounts for 60–70% of dementia cases, affecting millions of people in the aging population worldwide [1]. The hallmarks of AD include excessive accumulation of β-amyloid (Aβ) and intracellular neurofibrillary tangles which cause neuronal death and cognitive impairments. Two forms of AD, sporadic (i.e., late-onset) and familial (i.e., early-onset), comprise more than 95% and less than 5% of AD cases, respectively. Although the etiology of sporadic AD is largely unknown, impaired metabolic parameters including glycemic dysregulation and obesity are correlated with the development of AD-related pathology [2]. Hyperglycemia and obesity induced by a high fat diet (HFD) have been shown to exacerbate the amyloid pathology and cognitive impairment of AD transgenic mice and human [3,4,5]. Conversely, AD-related pathology leads to augmented obesity and glycemic dysregulation [4,6]. Therefore, ours and the other’s studies suggest that HFD and AD-related pathology reciprocally augment metabolic stresses and cognitive impairments [7].

Leptin, released by adipose tissue in proportion to fat mass is involved in regulating long-term food intake and energy homeostasis [8]. Hypothalamic leptin-receptor expressing neurons are mainly localized in the arcuate nucleus (ARC), ventromedial hypothalamus (VMH), and dorsomedial hypothalamus (DMH) in the mediobasal hypothalamus (MBH) [9,10]. The binding of leptin onto leptin receptor induces the phosphorylation of signal transducer and activator of transcription 3 (STAT3) in MBH. HFD-induced obesity induces neuroinflammation and elevates the basal STAT3 phosphorylation in the ARC [11,12]. The sustained increase of pSTAT3 in the ARC attributes to dysfunctional energy homeostasis. Despite mounting evidence demonstrating the pathophysiological alterations present throughout the central nervous system in AD, it is still unclear how AD pathology augments the development of metabolic diseases [13]. Because the hypothalamus is the control center for the energy intake and expenditure, its dysfunction can result in the development of diabetes mellitus and obesity [14,15,16].

Structural atrophy, amyloid plaques, and neuronal loss in AD patients occur in the limbic system including the prefrontal cortex, hippocampus, and hypothalamus [17]. Deregulated hypothalamic functions were reported in AD patients [18]. Consistently, impaired hypothalamic functions such as alternate feeding behaviors and excess food intake are reported in two AD transgenic mice models, 3xTgAD and Tg2576 mice [19,20]. 3xTgAD mice consume more food and are insensitive to cholecystokinin, a potent satiety factor [19]. Prior to the presence of the amyloid pathology in the hypothalamus, increased food intake and decreased proopiomelanocortin and neuropeptide Y neurons of 3xTgAD mice are observed [21]. Excess caloric intake is also observed in HFD-fed Tg2576 mice [20]. The chronic central perfusion of Aβ in HFD wildtype mice attenuates hypothalamic leptin sensitivity during feeding inhibition and leptin-induced pSTAT3 [22]. These studies suggest that dysfunctional hypothalamus in AD transgenic mice or Aβ-perfused HFD wildtype mice leads to excessive food intake and attenuated leptin signaling.

Although mounting evidence suggests that the hypothalamus is vulnerable in AD patients and transgenic mice, the molecular mechanisms underlying hypothalamus deregulation in AD remain unclear. In this study, we hypothesized that AD-related pathology augments HFD-induced metabolic stresses through the attenuation of hypothalamic leptin signaling and insulin signaling. We demonstrated that APP/PS1 transgenic mice exhibited worsening of HFD-induced metabolic stresses, via desensitization of hypothalamic leptin signaling and impaired peripheral insulin sensitivity.

## 2. Results

### 2.1. Alzheimer’s Disease-Related Pathology Interacts with High Fat Diet in Aggravating the Glucose Intolerance and Insulin Resistance of APPswe/PS1dE9 Transgenic Mice

No difference in the weight gain and glucose level was observed between wild type (WT) and APP/PS1 transgenic (AD) mice after 23- or 32-week HFD treatment in previous studies [3,23]. Our study has suggested that the duration of HFD treatment and age-related AD pathology of AD mice determine their individual impact on body weight gain and hyperglycemia [4]. To reveal the impact of AD-related pathology on the obesity and glycemic regulation, the fasting glucose levels of WT and AD mice were measured at 6, 11, 17, and 28 weeks after HFD treatment. After 11 weeks on HFD, the glucose level of AD mice was significantly higher than that of WT mice, but not after 6 or 17 weeks on HFD (Supplementary Materials Figure S1). Therefore, the further studies on the impact of AD-related pathology on glucose homeostasis were conducted after 10–12 weeks on HFD.

**Figure 1:**
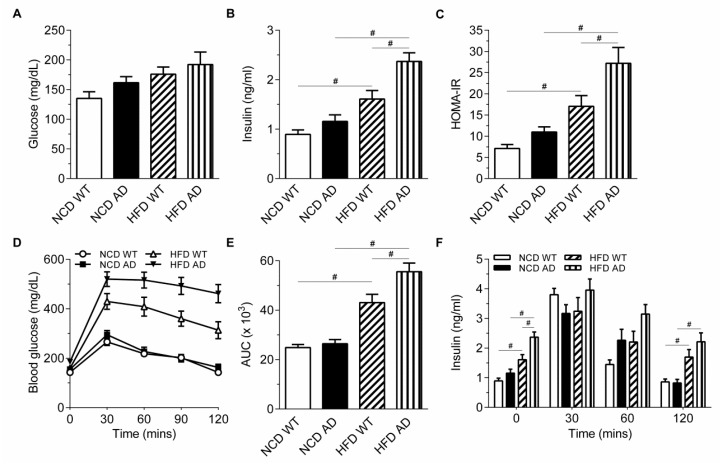
Impaired glycemic regulation is aggravated by AD-related pathology. (**A**,**B**) The level of glucose and insulin of NCD WT, NCD AD, HFD WT, and HFD AD mice measured at 10 weeks after the dietary switch (*n* = 7/group). (**C**) HOMA-IR was calculated (*n* = 7/group). (**D**,**E**) Glucose tolerance test was performed 10 weeks after the dietary manipulation and the area under curve (AUC) was calculated (*n* = 11/group). (**F**) The levels of insulin were measured at the indicated time points after glucose injection (*n* = 6–7/group). Data are expressed as the mean ± SEM. At least three independent experiments were performed. Statistical differences between groups were labeled with # (*p* < 0.05), determined by one-way ANOVA and Tukey’s HSD post-hoc tests.

The insulin level of HFD AD mice was elevated after 10 weeks on HFD while the glucose levels of HFD WT and HFD AD mice were comparable after 6 h fasting (Figure 1A,B). Homeostasis model assessment insulin resistance (HOMA-IR), an indirect measure of insulin resistance, suggested that augmented insulin resistance existed in HFD AD mice (Figure 1C). The glucose tolerance test (GTT) showed that an elevated glucose level was sustained in HFD AD mice after glucose challenge (Figure 1D,E). Our data suggested that the insulin resistance of HFD AD mice was worsened compared with normal chow diet (NCD) WT, NCD AD, and HFD WT mice. Regardless of the elevated fasting insulin of HFD AD mice at time zero, the peak level of insulin after 30 min glucose challenge was not significantly different among the four groups, indicating that the insulin secretion by pancreatic beta cells was unaffected in HFD AD mice (Figure 1F).

**Figure 2:**
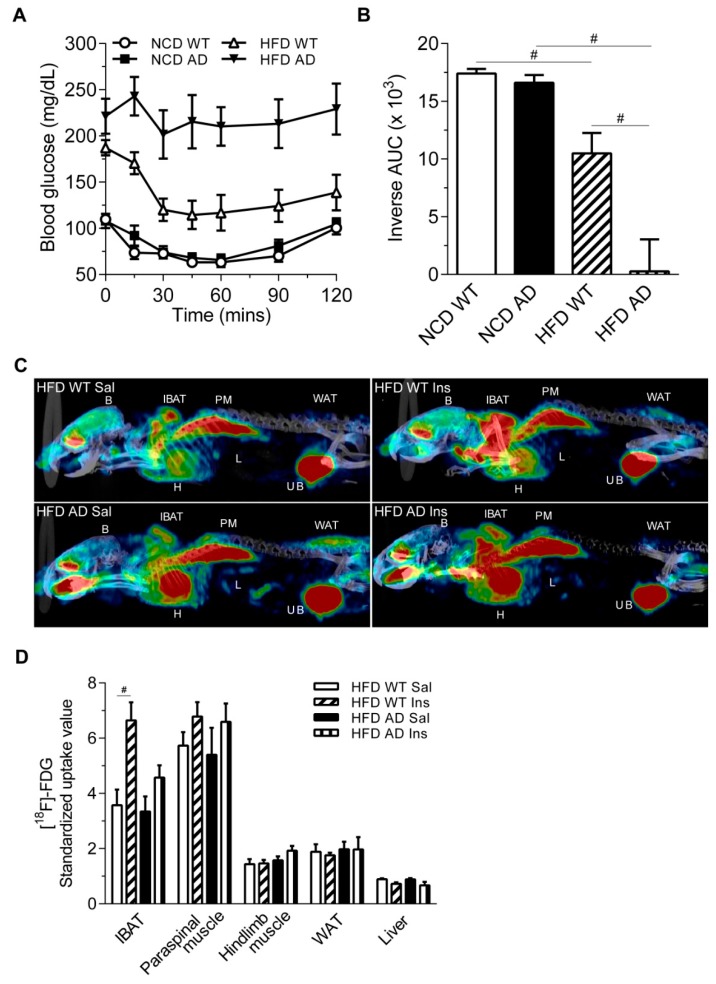
Insulin resistance and compromised insulin-induced glucose uptake in the interscapular brown adipose tissue of HFD AD mice. (**A**) The glucose level in response to insulin injection of NCD WT, NCD AD, HFD WT, and HFD AD mice was measured in the insulin tolerance test (*n* = 9/group). (**B**) The inverse area under the curve (AUC) was calculated (*n* = 9/group) (**C**) Representative [^18^F]-FDG PET/CT images of HFD WT and AD mice injected with the vehicle, saline (Sal) or insulin (Ins) at 12 weeks after the dietary switch. IBAT, interscapular brown adipose tissue; WAT, white adipose tissue; B, brain; L, liver; H, heart; PM, paraspinal muscle; UB, urinary bladder. (**D**) The standardized [^18^F]-FDG uptake value was calculated (*n* = 3–5/group). Data are expressed as the mean ± SEM. At least three independent experiments were performed. Statistical differences between groups were labeled with # (*p* < 0.05), determined by one-way ANOVA and Tukey’s HSD post-hoc tests.

The insulin tolerance test (ITT) showed that the glucose level of HFD AD mice was sustained after insulin injection, suggesting that AD mice were insensitive to insulin challenge after 12 weeks on HFD (Figure 2A,B). Two-way analysis of variance (ANOVA) revealed an interaction between the APP/PS1 genotype and HFD on the GTT and ITT (Table 1). [^18^F]-2-fluoro-2-deoxy-d-glucose ([^18^F]-FDG) positron emission tomography (PET) was used to identify insulin responsive tissues that were vulnerable to HFD and AD-related pathology. Insulin-induced [^18^F]-FDG uptake was observed in the interscapular brown adipose tissue (IBAT) of HFD WT mice, but not that of HFD AD mice (Figure 2C,D and Table 2). Attenuated insulin-induced glucose uptake by IBAT of HFD AD mice may contribute to the sustained glucose levels in the GTT and ITT. Nevertheless, the insulin-induced [^18^F]-FDG uptake by muscles, white adipose tissue, and liver was not observed in either HFD WT or AD mice.

### 2.2. The Obesity of High Fat Diet APPswe/PS1dE9 Transgenic Mice Is Exacerbated

**Figure 3:**
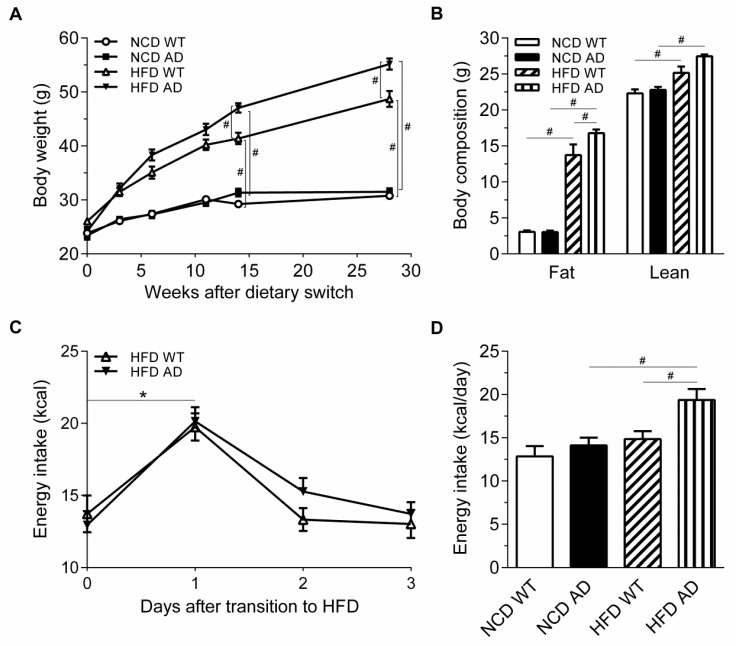
Excess energy intake contributes to the increased susceptibility to HFD-induced obesity in AD mice. (**A**) Body weight curves of NCD WT, NCD AD, HFD WT, and HFD AD mice (*n* = 18–19/group). (**B**) Body composition of the four groups was measured by Time-Domain Nuclear Magnetic Resonance (*n* = 5–6/group). (**C**) Energy intake of WT and AD mice on the first three days after the dietary switch from NCD to HFD (*n* = 10/group). (**D**) Daily energy intake of four groups was measured at 23 weeks after the dietary manipulations (*n* = 6–9/group). Data are expressed as the mean ± SEM. At least three independent experiments were performed. Statistical differences between groups were labeled with # (*p* < 0.05), determined by one-way ANOVA and Tukey’s HSD post-hoc tests (**A**,**B**,**D**). Statistical differences between groups were labeled with * (*p* < 0.05), determined by unpaired Student’s *t*-tests (**C**).

We next tested whether the AD-related pathology exacerbated HFD-induced body weight gain and food intake. Our data demonstrated that the body weight gain of AD mice was significantly higher than that of WT mice after 14 weeks on HFD. In contrast, no difference was observed between NCD WT and AD mice (Figure 3A). Body composition measurement indicated that the fat mass, but not lean mass, was higher in HFD AD mice (Figure 3B).

Normally, the body automatically adjusts the total calorie intake according to the dietary energy density. Since both excessive energy intake and reduced energy expenditure may lead to weight gain, we next measured the food intake and metabolic rates of mice fed with HFD. A previous study showed that excess energy intake of WT mice was elicited when the diet was switched from NCD to HFD [24]. Consistently, hyperphagia was observed in both WT and AD mice on the first day of switching from NCD to HFD, and the daily calorie intake returned to the baseline level on the third day (Figure 3C). The daily calorie intake of WT mice returned to baseline after 23 weeks on HFD; however, this appetite adjustment was impaired in AD mice, and these mice still consumed significantly more calories than the basal calorie intake after the dietary switch (Figure 3D).

### 2.3. The Basic Metabolic Rates of High Fat Diet Wild Type and High Fat Diet APPswe/PS1dE9 Transgenic Mice Are Comparable

**Figure 4:**
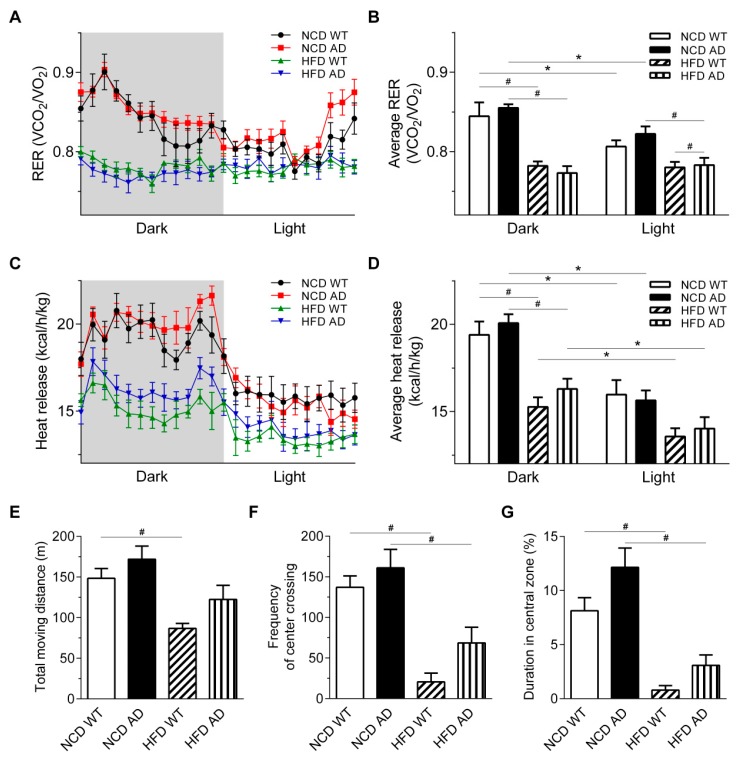
Metabolic rate and mobility of HFD AD and WT mice are comparable. (**A**) The RER of NCD WT, NCD AD, HFD WT, and HFD AD mice was calculated using the oxygen consumption (VO_2_) and carbon dioxide release (VCO_2_) measured by indirect calorimetry (*n* = 5–6/group). (**B**) Average RER during the light and dark cycle was calculated. (**C**) The heat release in the light and dark cycle was calculated (*n* = 5–6/group). (**D**) Average heat production during the light and dark cycle was calculated. (**E**–**G**) Total moving distance, frequency of center crossing, and the percentage of time spent in the center zone were compared at 28 weeks after the dietary switch (*n* = 7/group) in the open field test. Data are expressed as the mean ± SEM. At least three independent experiments were performed. Statistical differences between groups were labeled with # (*p* < 0.05), determined by one-way ANOVA and Tukey’s HSD post-hoc tests (**B**,**D**,**E**–**G**). Statistical differences between groups were labeled with * (*p* < 0.05), determined by paired Student’s *t*-tests (**B**,**D**).

The exacerbated body weight gain of HFD AD mice can be due to the reduced metabolic rate in addition to increased food intake; therefore, the oxygen consumption (VO_2_) and carbon dioxide release (VCO_2_) of the four groups were measured (Supplementary Materials Figure S2). The respiratory exchange ratio (RER), an indirect indicator of fuel utilization, was calculated by dividing VO_2_ by VCO_2_. The RER of NCD WT and AD mice was close to 1 in the dark cycle, indicating that NCD groups underwent greater carbohydrate utilization. The RER of HFD groups was closer to 0.7 in the dark cycle, indicating that HFD WT and AD mice underwent greater fatty acid utilization (Figure 4A). The average RER of the light and dark cycle of NCD groups was significantly different, suggesting that the diurnal regulation of the average RER was intact in NCD groups. In patients with diabetes, the RER is often correlated with impaired diurnal-regulated fuel switching between carbohydrate and fatty acid utilization [25]. Consistently, the average RER of HFD groups in the light and dark cycle was not different, suggesting that the diurnal regulation of the average RER was impaired in HFD groups (Figure 4B).

The heat release, an index of energy expenditure, was higher in the dark cycle than in the light cycle for all four groups (Figure 4C). However, the heat release was lower in HFD groups than in NCD groups in the dark cycle, suggesting that the energy expenditure of HFD groups was lower than that of NCD groups (Figure 4C,D). Nonetheless, the heat release by HFD WT and AD mice was comparable. Two-way ANOVA revealed an interaction between APP/PS1 genotype and HFD on the body weight and fat mass (Table 1).

To investigate whether the exacerbated weight gain of HFD AD mice could be partly attributed to reduced locomotor activity, the open field test was performed. The number of central crossings in the open field was evaluated as an index of anxiety. The total moving distance, frequency of the center crossing, and duration in the center zone were comparable between HFD groups, suggesting that locomotor activity and extent of anxiety of HFD WT and AD mice were similar (Figure 4E–G). Therefore, the excessive body weight gain of HFD AD mice was contributed by the increased energy intake.

### 2.4. Leptin-Induced Feeding Suppression in High Fat Diet APPswe/PS1dE9 Transgenic Mice Is Obliterated

**Figure 5:**
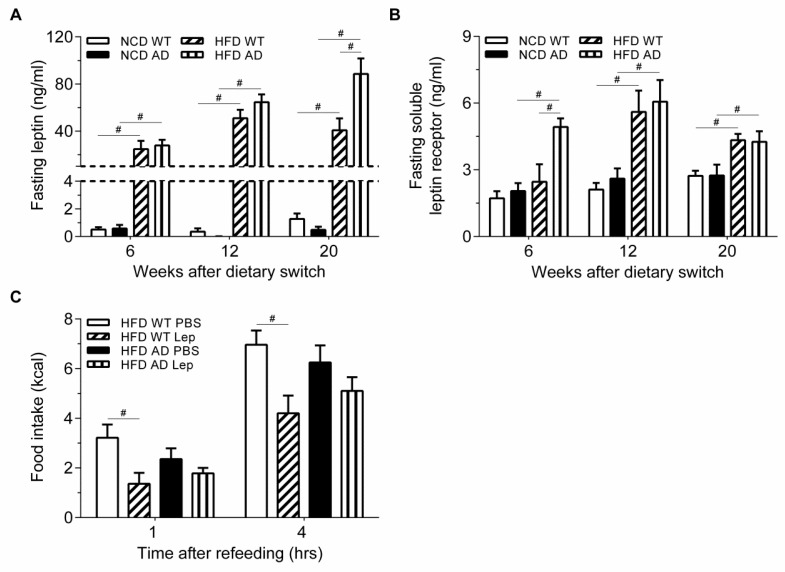
Leptin fails to suppress the energy intake of HFD AD mice, but not HFD WT mice. (**A**,**B**) Levels of fasting leptin and soluble leptin receptor of NCD WT, NCD AD, HFD WT, and HFD AD mice were measured (*n* = 7/group). (**C**) The cumulative energy intake of the four groups was measured after an i.p. injection of leptin (Lep) or the vehicle (PBS) (*n* = 7–8/group). Data are expressed as the mean ± SEM. At least three independent experiments were performed. Statistical differences between groups were labeled with # (*p* < 0.05), determined by one-way ANOVA and Tukey’s HSD post-hoc tests.

The exacerbated hyperphagia, body weight gain, and increased fat mass of HFD AD mice compared with HFD WT mice prompted us to test whether HFD AD mice have abnormal leptin signaling. After 6 weeks on HFD, hyperleptinemia was induced; however, the level of leptin was comparable in HFD WT and AD mice (Figure 5A). After 20 weeks on HFD, the level of leptin was significantly higher in HFD AD mice. Two-way ANOVA revealed an interaction between APP/PS1 genotype and HFD on the level of leptin (Table 1).

The soluble leptin receptor lacking a transmembrane domain can bind leptin and thus reduces the level of effective leptin in the bloodstream [26]. Our data demonstrated that the serum level of soluble leptin receptor was higher in HFD groups than in NCD groups. Furthermore, the serum level of soluble leptin receptor of AD mice was even higher than that of WT mice after 6 weeks on HFD. Elevated soluble leptin receptor may prevent leptin transport into the brain and contribute to the hyperphagia and increased body weight of HFD AD mice (Figure 5B).

The excess food intake with elevated leptin of HFD AD mice suggested that they were more leptin insensitive (Figure 3D and Figure 5A). To test this hypothesis, HFD WT and AD mice were injected with leptin to assess whether exogenous leptin can suppress energy intake. Exogenous leptin suppressed the energy intake of WT mice, but not AD mice after 23 weeks on HFD, suggesting that the leptin sensitivity of HFD AD mice was further attenuated (Figure 5C). Thus, the obese phenotype in HFD AD mice might result from attenuated leptin signaling in its target organs where leptin actively modulates energy homeostasis.

### 2.5. Leptin Signaling in the Arcuate Nucleus Is Attenuated in High Fat Diet APPswe/PS1dE9 Transgenic Mice

Next, we examined leptin signaling in the MBH of NCD WT and AD mice by comparing the number of pSTAT3-positive cells after leptin or PBS injection at an age of 9.5 months to match the age of HFD groups after 28 weeks of dietary manipulations (Supplementary Materials Figure S3A–C). The numbers of leptin-induced pSTAT3-positive cells in the ARC, VMH, and DMH of NCD WT and AD mice were comparable, suggesting that the leptin signaling of NCD AD mice remained intact (Supplementary Materials Figure S3D). HFD has been shown to increase the number of basal pSTAT3-positive cells in the MBH of WT mice [27]. Consistently, we observed the elevated basal pSTAT3-positive cells in the MBH of WT and AD mice after 28 weeks on HFD (Supplementary Materials Figure S3E).

**Figure 6:**
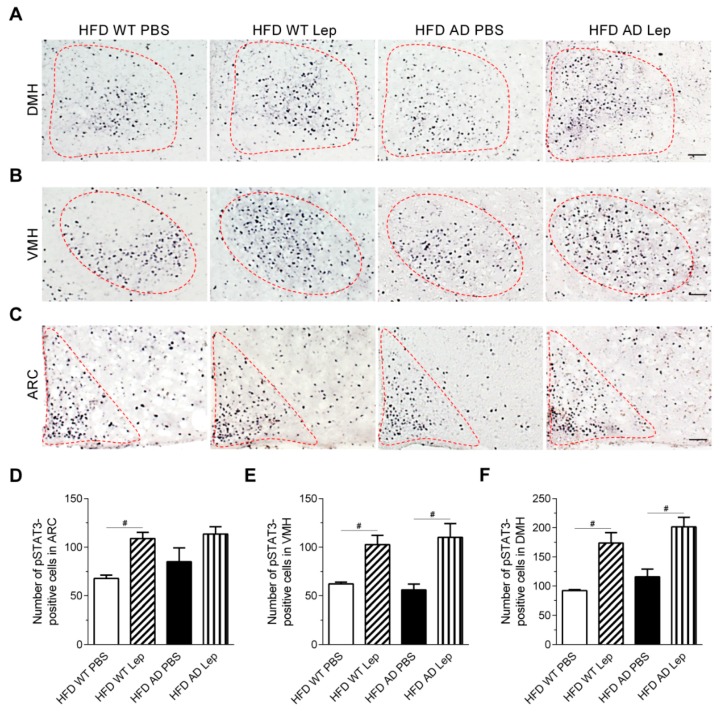
Leptin fails to induce STAT3 phosphorylation in the ARC of HFD AD mice. (**A**–**C**) Representative images of pSTAT3-positive cells in the ARC, VMH, and DMH of HFD WT and AD mice after the injection of leptin (Lep) or the vehicle (PBS). The third ventricle is on the left of each panel. The red dotted line defined the area of the DMH (**A**), VMH (**B**), and ARC (**C**) in which numbers of pSTAT3-positive cells were quantified. Scale bar, 55 µm (**A**) and 50 µm (**B**,**C**). (**D**–**F**) The numbers of pSTAT3-positive cells in the ARC, VMH, and DMH were quantified (*n* = 3–5/groups). Data are expressed as the mean ± SEM. At least three independent experiments were performed. Statistical differences between groups were labeled with # (*p* < 0.05), determined by one-way ANOVA and Tukey’s HSD post-hoc tests.

Since hyperphagia in HFD AD mice might be caused by attenuated leptin signaling in the hypothalamus, we examined the number of pSTAT3-positive cells in the MBH of HFD groups, after leptin or PBS injection (Figure 6A–C). Leptin increased the number of pSTAT3-positive cells in the ARC of HFD WT, but not AD mice after 28 weeks on HFD (Figure 6D). Nevertheless, the number of leptin-induced pSTAT3-positive cells in the VMH and DMH of HFD WT and AD mice was comparable (Figure 6E,F). This suggested that the leptin signaling was attenuated in the ARC of HFD AD mice specifically. Two-way ANOVA revealed that leptin had a main effect on the number of pSTAT3-positive cells in the ARC, VMH, and DMH of HFD groups (Table 2).

### 2.6. Elevated Inflammation and Aβ in the Circulation and Cortex, but Not Hypothalamus of High Fat Diet APPswe/PS1dE9 Transgenic Mice

Since our data suggested that hypothalamic regulation of energy homeostasis of HFD AD mice was defective, we next investigated whether AD-related pathology including the Aβ accumulation and reactive astrocytes was present in the MBH of AD mice. The β-sheet structure of Aβ aggregates was stained by (*trans*, *trans*)-1-bromo-2,5-bis-(3-hydroxycarbonyl-4-hydroxy) styrylbenzene (BSB) to detect the presence of senile plaques. Anti-glial fibrillary acidic protein (anti-GFAP) antibody was used to detect reactive astrocytes. Senile plaque was not present in the MBH of four groups at the age of 9.5 months (Supplementary Materials Figure S4A). Furthermore, the intensity of GFAP in the ARC was not different between the four groups (Supplementary Materials Figure S4B). Consistently, Aβ ELISA showed that the levels of sodium dodecyl sulfate (SDS)-soluble and SDS-insoluble Aβ species in the hypothalamus of four groups were also undetectable (*n* = 5–6/group). Our data suggested that hypothalamic dysregulation of energy homeostasis was elicited prior to the presence of Aβ accumulation in the hypothalamus of HFD AD mice.

**Figure 7:**
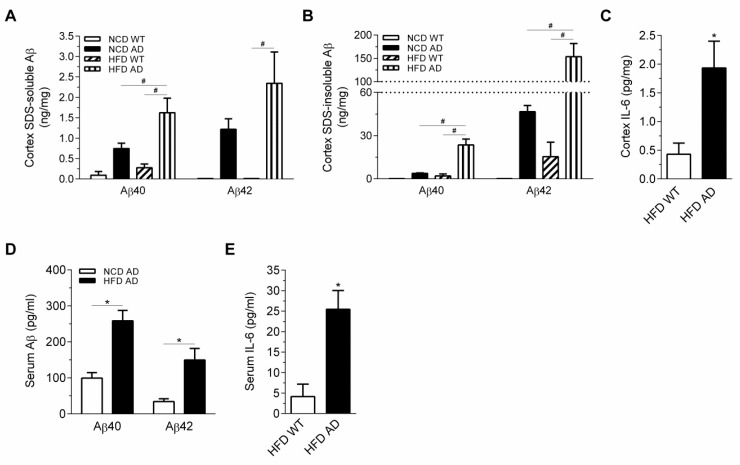
Neuroinflammation and peripheral inflammation of HFD AD mice are exacerbated. (**A**,**B**) The cortical levels of Aβ40 and Aβ42 of four groups were measured (*n* = 5–6/group). (**C**) The cortical levels of IL-6 of HFD groups (*n* = 6–8/group) were measured. (**D**) The serum levels of Aβ40 and Aβ42 in NCD and HFD AD mice were measured (*n* = 6–8/group). (**E**) The serum level of IL-6 in HFD WT and AD mice was measured (*n* = 6–8/group). Data are expressed as the mean ± SEM. At least three independent experiments were performed. Statistical differences between groups were labeled with # (*p* < 0.05), determined by one-way ANOVA and Tukey’s HSD post-hoc tests (**A**,**B**). Statistical differences between groups were labeled with * (*p* < 0.05), determined by unpaired Student’s *t*-tests (**C**–**E**).

Next, the burden of senile plaques and astrocytic reactivity of four groups in the cortex were examined (Supplementary Materials Figure S4C). GFAP intensity of HFD AD mice was significantly higher than that of HFD WT mice, suggesting that the neuroinflammation was worsened in HFD AD mice (Supplementary Materials Figure S4D). Reactive astrocytes in the vicinities of senile plaques in the cortex were also observed in NCD AD mice. The intensity of GFAP and the coverage of BSB-stained senile plaques in the cortex were comparable in HFD and NCD AD mice at the age of 9.5 months (Supplementary Materials Figure S4D,E). Since BSB staining only detected Aβ aggregates with β-sheet structure, Aβ ELISA was performed to detect the total account of Aβ aggregates with diverse conformations. The ELISA data showed that the cortical levels of SDS-soluble and SDS-insoluble Aβ were significantly elevated in HFD AD mice compared with NCD AD mice (Figure 7A,B). Therefore, HFD augmented cortical Aβ accumulation of AD mice.

The level of interleukin-6 (IL-6), a widely used inflammatory marker, was increased in the cortex of HFD AD mice compared with HFD WT mice (Figure 7C). Moreover, serum levels of Aβ and IL-6 were also higher in HFD AD mice than in NCD AD mice (Figure 7D,E). Upregulated STAT3 in 3xTgAD mice mediates astrocyte reactivity and contributes to IL-6 upregulation [28,29]. We next examined whether STAT3 was phosphorylated in the cortex of AD mice. The presence of nuclear pSTAT3 in GFAP-positive astrocyte and neurons of HFD AD mice suggested that the neuroinflammation was elevated compared with HFD WT mice (Supplementary Materials Figure S5). Taken together, our data indicated that the inflammatory context in the central nervous system and the peripheral of HFD AD mice was significantly elevated.

## 3. Discussion

The clinical symptoms of AD patients include alterations in glucose and energy homeostasis; however, the underlying mechanisms remain unclear [18]. The present study reveals that HFD-induced insulin resistance and hypothalamic leptin resistance are augmented by the AD-related pathology which can result in exacerbated weight gain and glycemic dysregulation. Our results further suggested that aggravated hypothalamic impairment of HFD AD mice was associated with elevated Aβ and IL-6 in the brain and circulation. Our study is the first study suggesting that exacerbated HFD-induced obesity and glycemic dysregulation in APP/PS1 transgenic mice are associated with impaired insulin and hypothalamic leptin signaling.

Previously, we showed that aging of AD mice aggravates the increase of senile plaques and neuroinflammation can mask the impact of HFD treatment. To prevent the masking of HFD on the impact of AD-related pathology on energy and glucose homeostasis, we monitored the metabolic index along the dietary manipulations and identified the optimal duration of HFD treatment and revealed the critical role of AD-related pathology in glycemic and energy homeostasis. Two-way ANOVA indicated that the glycemic dysregulation, body weight gain, hyperleptinemia, and increased fat tissue mass are attributed to the interaction between the diets and the APP/PS1 genotype (Table 1, *p* < 0.001). This strongly demonstrated that the interaction between HFD and AD-related pathology contributed to the hypothalamic dysregulation of energy hemostasis.

Leptin and insulin are two orexigenic hormones targeting the hypothalamus and mediate the autonomic control of glucose and energy homeostasis [30]. Therefore, insulin and leptin resistance of HFD AD mice lead to hyperleptinemia, hyperglycemia, and hyperphagia in a vicious cycle and ultimately the worsened metabolic stresses. Attenuated amplitudes of leptin-induced pSTAT3 in the ARC of HFD AD mice may lead to the reduced efficacy of leptin in suppressing food intake. Intracerebroventricular Aβ injection induces glucose intolerance and increased food intake [31]. Clarke et al. suggest that hypothalamic ER stress and inflammation induced by Aβ are critical for glucose deregulation. Long-term perfusion of Aβ attenuates the induction of pSTAT3 by leptin in HFD WT mice, suggesting that Aβ promotes hypothalamic leptin resistance and the body weight gain [22]. These studies indicate that exogenous Aβ impairs hypothalamic leptin signaling and glucose homeostasis. Circulating cytokines may contribute to the attenuated leptin signaling in HFD groups [27,32]. Furthermore, IL-6 also increases the level of pSTAT3 at ARC, VMH, and DMH [33]. Taken together, chronically elevated levels of diffusible Aβ and IL-6 in HFD AD mice in the circulating may blunt the insulin and leptin signaling which resulted in exacerbated metabolic alterations in addition to the disrupted limbic system.

The limbic system includes the hippocampus, limbic cortex, and hypothalamus; these brain regions are functionally interconnected and involved in the feeding behavior, and the interconnection of the limbic system can be disrupted during AD progression [34]. Our previous [^18^F]-FDG uptake showed that glucose usage was reduced in the hypothalamus, in addition to the cortex and hippocampus in HFD AD mice, suggesting that the neuronal activities throughout the limbic system were suppressed [4]. Therefore, the elevated neuroinflammation in the cortex and hippocampus may impair the hypothalamic function of HFD AD mice prior to the presence of hypothalamic amyloid pathology.

IBAT, muscles, and white adipose tissues are glucose sinks for glycemic homeostasis and insulin sensitivity [25,35]. IBAT is involved in energy homeostasis and thermoregulation during excess feeding and cold challenge [36]. When systematic insulin resistance is established after 13 weeks on HFD demonstrated by the glucose and insulin tolerance tests, attenuated insulin-induced glucose uptake IBAT of AD mice suggests that IBAT is more vulnerable to HFD under an AD genetic background. Whether exacerbated obesity of HFD AD mice resulted in impaired hypothalamic thermoregulation and less active IBAT in response to the cold challenge remains to be studied. It has been shown that IBAT is more resistant to HFD-mediated reduction in insulin-mediated glucose uptake as compared to skeletal muscle and white adipose tissue [37]. Consistently, our data also indicated that the FDG uptake by muscles and white adipose tissue of WT and AD mice was not stimulated by insulin, suggesting that muscle and white adipose tissue were insulin resistant after 13 weeks on HFD.

## 4. Materials and Methods

### 4.1. Animals

This study was conducted in strict accordance with the recommendations of the Guide for the Care and Use of Laboratory Animals of National Institutes of Health. The Institutional Animal Care and Use Committee at National Yang Ming University approved all the animal protocols (IACUC No: 1041251, 30 December 2015). APPswe/PS1dE9 (APP/PS1) transgenic mice (Mutant Mouse Resource and Research Center stock #034832) were purchased from Mutant Mouse Resource and Research Center at Jackson Laboratories and bred in-house. The mice were fed with a normal chow diet (NCD) with 13.6% kcal from fat (MFG, Oriental Yeast Co., Ltd., Tokyo, Japan) and water *ad libitum*. Mice were housed under controlled room temperature (24 ± 1 °C) and humidity (55–65%), with a 12 h dark cycle (19:00 to 07:00) and 12 h light cycle (07:00 to 19:00). More than three cohorts of male APP/PS1 transgenic (AD) and wild type (WT) littermates were randomly assigned to the dietary manipulations at the age of 10 weeks as described [4,6,22]. One group of male AD mice and WT siblings was switched to the high fat diet (HFD; with 60% kcal from fat; Research Diet #D12492, USA, New Brunswick, NJ, USA), and designated as HFD AD mice and HFD WT mice, respectively. The other groups of male APP/PS1 transgenic and WT mice were maintained on NCD designated as NCD AD mice and NCD WT mice, respectively. The biochemical index of the four experimental groups, NCD AD, NCD WT, HFD AD, and HFD WT mice, were measured at the indicated ages. Mice were sacrificed after 28-week dietary manipulations at the age of 9.5 months.

### 4.2. Glucose Tolerance Test and Insulin Tolerance Test

For the glucose tolerance test, mice were i.p. injected with 1.5 g/kg glucose after a 6 h fasting as described [38,39]. The levels of glucose and insulin were measured simultaneously. For the insulin tolerance test, mice were i.p. injected with insulin (0.75 unit/kg, Sigma-Aldrich, St. Louis, MO, USA) after a 6 h fasting [39]. The level of glucose was measured at the indicated time points.

### 4.3. In Vivo [^18^F]-2-fluoro-2-deoxy-d-glucose Positron Emission Tomography Experiment

After a 4 h fasting, HFD WT and HFD AD mice were subjected to an i.p. injection of [^18^F]-2-fluoro-2-deoxy-d-glucose ([^18^F]-FDG) after 13 weeks of dietary switch as described [40] with modifications. Briefly, 30 min after injection of [^18^F]-FDG, mice were i.p. injected with saline or insulin (0.75 unit/kg). After 20 min, mice were i.p. injected with pentobarbital (80 mg/kg) and underwent image acquisition using static positron emission tomography (PET) and a computed tomography scan (Sedecal, Madrid, Spain).

### 4.4. Measurement of Biochemical Parameters

The serum levels of glucose, insulin, leptin, and soluble leptin receptor were measured using blood samples collected from the tail vein. The level of insulin was determined using the homogeneous time resolved fluorescence detection method (Cisbio, Codolet, France); fluorescence intensity was detected at 620 and 665 nm using a SpectraMax M5 microplate reader (Molecular Devices, San Jose, CA, USA). The homeostasis model assessment for insulin resistance index (HOMA-IR) was calculated as fasting blood glucose (mM) × fasting insulin (U/mL)/22.5. The fasting levels of leptin and soluble leptin receptor were measured using ELISA kits (R&D Systems, Minneapolis, MN, USA). The fluorescence intensity at 450 nm was detected.

### 4.5. Body Composition and Metabolic Rate Analysis

Body composition of four groups after the 18-week dietary manipulations was measured using Time-Domain Nuclear Magnetic Resonance (TD-NMR) technology and indirect calorimetry was performed in the TSE metabolic cage environment (TSE Systems Inc., Chesterfield, MO, USA) by Mouse Clinics of Academia Sinica in Taiwan. The levels of O_2_ and CO_2_ in each cage of singly housed mice were recorded at 30 min intervals for 48 h and compared with reference air. Oxygen consumption (VO_2_) and carbon dioxide release (VCO_2_) were measured. Respiratory exchange ratio (RER), which is an indicator of the basal metabolic rate, was calculated as VCO_2_/VO_2_. Heat release was calculated as [CV × VO_2_ + CV × VCO_2_]/1000, where CV refers to the calorific value.

### 4.6. Measurement of Feeding Behavior

Mice were singly housed and acclimated with a food container (Oriental Yeast Co., Ltd., Tokyo, Japan) for 3 days. Daily food intake of WT and AD mice was assessed using a food container at the first 3 days after switching from NCD to HFD and after 23 weeks on HFD. Leptin-induced feeding suppression was performed as described [41] after 23 weeks on HFD. Briefly, mice were deprived of food from 02:30 for 18 h and were i.p. injected with leptin (3 mg/kg, R&D Systems, Minneapolis, MN, USA). After 30 min, food pellets were placed into the food container and the 1 h and 4 h accumulated food intake was measured.

### 4.7. Assessment of Locomotor Activity in Open Field

Mice were placed in Plexiglass chambers (40 × 40 × 37 cm) and video-recorded for 60 min. The area of the central zone was defined as 10 cm away from the walls. The total moving distance, times of center crossing, and duration in the center zone were analyzed by EthoVision software (Noldus, Leesburg, VA, USA).

### 4.8. Immunohistochemistry and Senile Plaque Staining

WT and AD mice after 28 weeks on HFD were injected with PBS or leptin (4 mg/kg) after 16 h fasting as described [42]. After 45 min, mice were perfused with 4% paraformaldehyde and brain tissues were cryoprotected with sucrose solutions. Brain sections with a 20-µm thickness spanning from Bregma −1.9 mm to Bregma −2.06 mm were incubated with anti-pSTAT3 (at Tyr705) antibody (1:1000, Cell Signaling, Danvers, MA, USA) in 0.1% PBST overnight, and washed. The brain slices were then incubated with biotinylated secondary antibody (1:1000, Jackson ImmunoResearch Laboratories, West Grove, PA, USA), followed by the coloring reaction. Images were obtained using an Olympus DP73 microscope. The areas of the ARC, VMH, and DMH were defined by the clustered nuclei stained with DAPI or Neutral Red prior to pSTAT3 immunohistochemical staining. The number of pSTAT3-positive cells was quantified using ImageJ software, version 1.38x (The National Institutes of Health, Bethesda, MD, USA) after converting color images into grayscale images. The pSTAT3-positive cell numbers were averaged from at least three anatomically matched brain slices of HFD WT and AD mice with PBS or leptin injection. The cells with the intensity of pSTAT3above the threshold set at 0–100 of ImageJ software were counted as pSTAT3-positive cells. In addition, the circularity of pSTAT3-positive cells was set at 0.7–1, and the size of the signal was set at larger than 5 pixels to avoid dot-like signals detected in the neuronal process of HFD groups. A visual inspection was performed on each brain slice after the quantification performed by ImageJ.

To visualize senile plaques and reactive astrocytes in the hypothalamus and cortex, brain slices were incubated with 0.01% BSB as described [43], then incubated with anti-GFAP antibody (Sigma) followed by Alexa Fluor 594-labeled secondary antibody (1:500, Life technologies, Carlsbad, CA, USA). The relative fluorescence intensity of GFAP and BSB in the cingulate cortex was quantified using ImageJ software.

### 4.9. Aβ Measurement

The cerebral cortex and hypothalamus were homogenized in PBS containing 0.5% SDS and 0.5% Triton X-100 as described [4]. Following sonication and centrifugation, the supernatant was designated as the SDS-soluble fraction. The pellet was suspended in 3 M guanidine-HCl and sonicated. Following centrifugation, the supernatant was designated as the SDS-insoluble fraction. The level of Aβ in the SDS-soluble, SDS-insoluble fractions, and the serum was measured using Aβ ELISA kits (Invitrogen, Carlsbad, CA, USA).

### 4.10. Statistical Analysis

Statistical analyses were performed using GraphPad Prism (GraphPad Software, version 6.01, San Diego, CA, USA) and SPSS (IBM, Armonk, NY, USA) software. All values are reported as the mean ± standard error of the mean (SEM). All experiments were performed more than three times. Data of the four groups were analyzed using one-way analysis of variance (ANOVA) followed by Tukey’s honest significant difference post-hoc test. # (*p* < 0.05) was considered significant. The comparisons of Aβ, IL-6, GFAP/BSB and energy intake between two groups were performed using unpaired Student’s *t*-tests. The comparisons of averaged RER and heat release between two groups were performed using paired Student’s *t*-tests. * (*p* < 0.05) was considered significant. Two-way ANOVA (general linear model) was used to analyze the interaction between dietary manipulations and genotypes on the insulin sensitivity, energy homeostasis, basic metabolic rate, level of leptin and soluble leptin receptor, shown in Table 1, and treatments (insulin or leptin) and genotypes on [^18^F]-FDG uptake, energy intake and the pSTAT3-positive cells, shown in Table 2.

## 5. Conclusions

This study revealed that impaired leptin and insulin signaling mediated exacerbated obesity and glucose dysregulation through the interaction of the APP/PS1 genotype and HFD. The pathophysiological events discovered in our HFD-fed APP/PS1 transgenic mouse model indicate that metabolic biomarkers can be used to monitor the response to therapeutic interventions at early disease stages. Further, this study also helps raise public awareness that a neurodegenerative condition such as AD might exacerbate a patient’s metabolic condition.

## Figures and Tables

**Table 1:** Two-way analysis of variance (ANOVA) of the interactions between the dietary manipulations and genotypes. Two-way ANOVA revealed an interaction between diets and genotypes on the body weight, fat mass, and insulin sensitivity. The diet and genotype exerted main effects on the daily energy intake and lean mass.

Metabolic Index	Interaction F_interaction_ *p* Value	Main Effect F_group_ *p* Value		Simple Main Effect F_group_ *p* Value			
		Diet	Genotype	NCD	HFD	WT	AD
**Insulin**	2.924 *p* = 0.100	43.499 *p* < 0.001	12.236 *p* < 0.005				
**HOMA-IR**	1.726 *p* = 0.201	30.080 *p* < 0.001	8.625 *p* < 0.01				
**GTT**	4.287 *p* < 0.05			0.565 *p* = 0.461	6.674 *p* < 0.05	25.876 *p* < 0.001	56.279 *p* < 0.001
**ITT**	7.831 *p* < 0.01			1.102 *p* = 0.309	9.742 *p* < 0.01	14.745 *p* < 0.005	33.102 *p* < 0.001
**Body weight**	8.403 *p* < 0.01			0.705 *p* = 0.407	13.327 *p* < 0.005	130.958 *p* < 0.001	372.177 *p* < 0.001
**Fat mass**	4.624 *p* < 0.05			0.008 *p* = 0.932	3.896 *p* = 0.084	62.659 *p* < 0.001	757.592 *p* < 0.001
**Lean mass**	2.677 *p* = 0.119	45.369 *p* < 0.001	6.142 *p* < 0.05				
**Daily energy intake**	2.244 *p* = 0.145	11.138 *p* < 0.005	7.124 *p* < 0.05				
**Hourly respiratory exchange ratio**							
Day	0.585 *p* = 0.454	14.923 *p* < 0.005	1.249 *p* = 0.278				
Night	0.793 *p* = 0.385	43.524 *p* < 0.001	0.005 *p* = 0.946				
**Hourly heat release**							
Day	0.350 *p* = 0.561	9.152 *p* < 0.01	0.009 *p* = 0.927				
Night	0.084 *p* = 0.775	40.638 *p* < 0.001	1.886 *p* = 0.186				
**Leptin**	8.584 *p* < 0.01			2.967 *p* = 0.111	8.317 *p* < 0.05	15.082 *p* < 0.005	44.696 *p* < 0.001
**Soluble leptin receptor**	0.013 *p* = 0.909	16.322 *p* < 0.001	0.006 *p* = 0.937				

HOMA-IR, homeostasis model assessment insulin resistance; GTT, glucose tolerance test; ITT, insulin tolerance test.

**Table 2:** Two-way analysis of variance (ANOVA) of the interaction between treatments and genotypes. Two-way ANOVA analysis revealed that insulin had a main effect on insulin-induced glucose uptake by interscapular brown adipose tissue. Leptin had a main effect on the energy intake and pSTAT3 induction.

Impact of Insulin and Leptin	Interaction F_interaction_ *p* Value	Main Effect F_group_ *p* Value	
		Treatment	Genotype
**Insulin-induced [^18^F]-FDG uptake**			
IBAT	2.121 *p* = 0.171	11.537 *p* < 0.01	3.291 *p* = 0.095
**Leptin inhibition of energy intake**			
1 h energy intake	2.374 *p* = 0.135	8.542 *p* < 0.01	0.269 *p* = 0.609
4 h energy intake	1.595 *p* = 0.218	9.388 *p* < 0.01	0.021 *p* = 0.887
**Leptin-induction of pSTAT3**			
ARC	0.469 *p* = 0.508	14.602 *p* < 0.005	1.433 *p* = 0.256
VMH	0.805 *p* = 0.389	39.044 *p* < 0.001	0.009 *p* = 0.928
DMH	0.029 *p* = 0.868	48.619 *p* < 0.001	4.599 *p* = 0.055

IBAT, interscapular brown adipose tissue; ARC, arcuate nucleus; VMH, ventromedial hypothalamus; DMH, dorsomedial hypothalamus.

## Supplementary Materials

Supplementary materials can be found at http://www.mdpi.com/1422-0067/19/8/2333/s1.

## Abbreviations

Aβ	β-amyloid
AD	Alzheimer’s disease
ANOVA	analysis of variance
APP	amyloid precursor protein
ARC	arcuate nucleus
BSB	(*trans*, *trans*)-1-bromo-2,5-bis-(3-hydroxycarbonyl-4-hydroxy) styrylbenzene
DMH	dorsomedial hypothalamus
GFAP	glial fibrillary acidic protein
HFD	high-fat diet
HOMA-IR	homeostasis model assessment for insulin resistance index
HSD	honestly significant difference
IBAT	interscapular brown adipose tissue
IL-6	interleukin-6
i.p.	intraperitoneally
NCD	normal chow diet
RER	respiratory exchange ratio
SEM	standard error of mean
T2DM	type 2 diabetes mellitus
VCO_2_	carbon dioxide release
VMH	ventromedial hypothalamus
VO_2_	oxygen consumption
WT	wildtype
