# Supplementary material for: Augmented Insulin and Leptin Resistance of High Fat Diet-Fed APPswe/PS1dE9 Transgenic Mice Exacerbate Obesity and Glycemic Dysregulation
Source: Int J Mol Sci. 2018 Aug 8;19(8):2333. doi: 10.3390/ijms19082333 (PMC6121904; doi:10.3390/ijms19082333)
Supplement: Supplementary file 1 [file ijms-19-02333-s001.pdf]

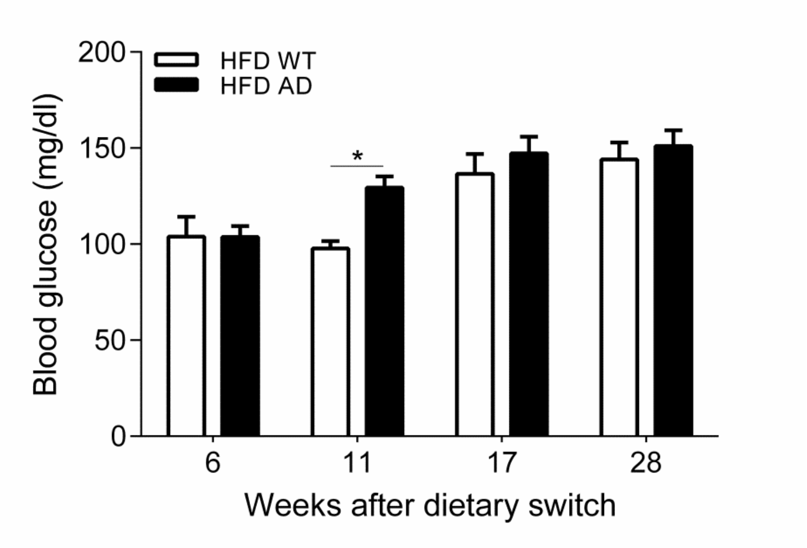

Supplementary Figure S1. The impact of AD-related pathology on the glycemic regulation. The fasting glucose was measured at indicated durations after the diet switch from NCD to HFD after 16-h fasting (*n* = 11/group). The glucose level of AD mice was significantly higher than WT mice after 11 weeks on HFD. Data was expressed as mean ± SEM. At least three independent experiments were performed. Statistically different between groups were labeled with * ($p < 0.05$), determined by unpaired Student's t-tests.

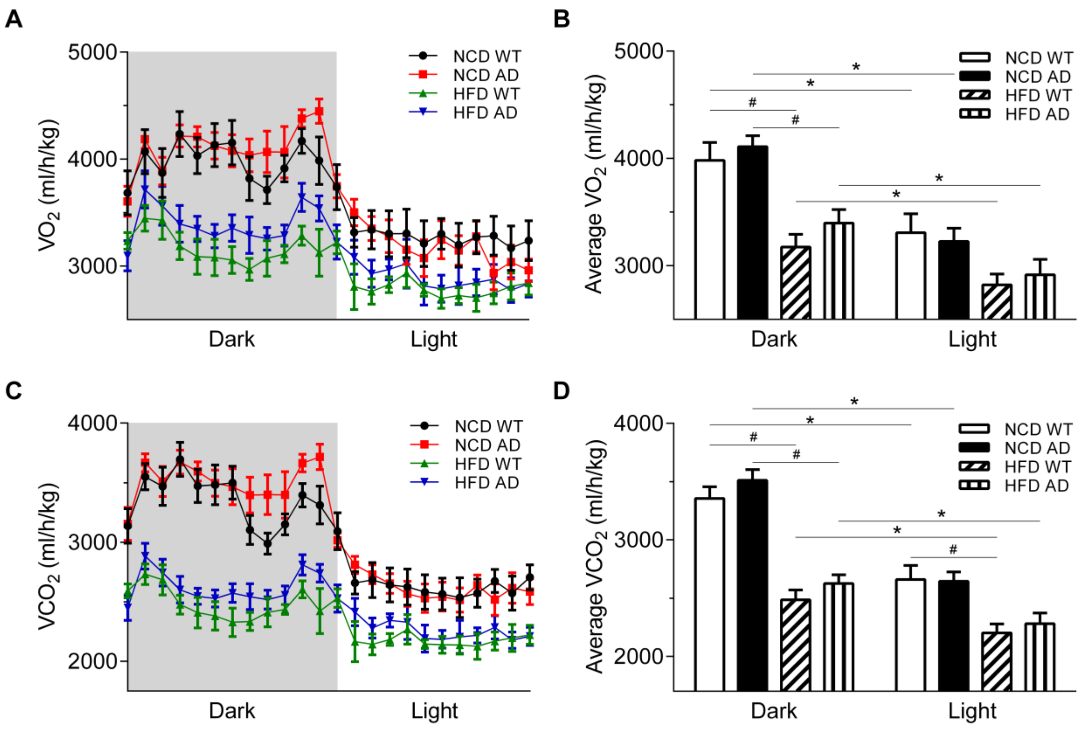

Supplementary Figure S2. AD-related pathology elicits little effect on the basic metabolic rate compared with HFD treatment. (**A,B**) Oxygen consumption (VO_2) of four groups was measured and the average VO_2 in the dark and light cycle was calculated (*n* = 5-6/group). (**C,D**) Carbon dioxide release (VCO_2) measured and the average VCO_2 in the dark and light cycle was calculated (*n* = 5-6/group). Data was expressed as mean ± SEM. At least three independent experiments were performed. Statistically different between groups were labeled with # ($p < 0.05$), determined by

one-way ANOVA and Tukey's HSD post-hoc tests (**B,D**). Statistically different between groups were labeled with * ($p < 0.05$), determined by paired Student's t-tests (**B,D**).

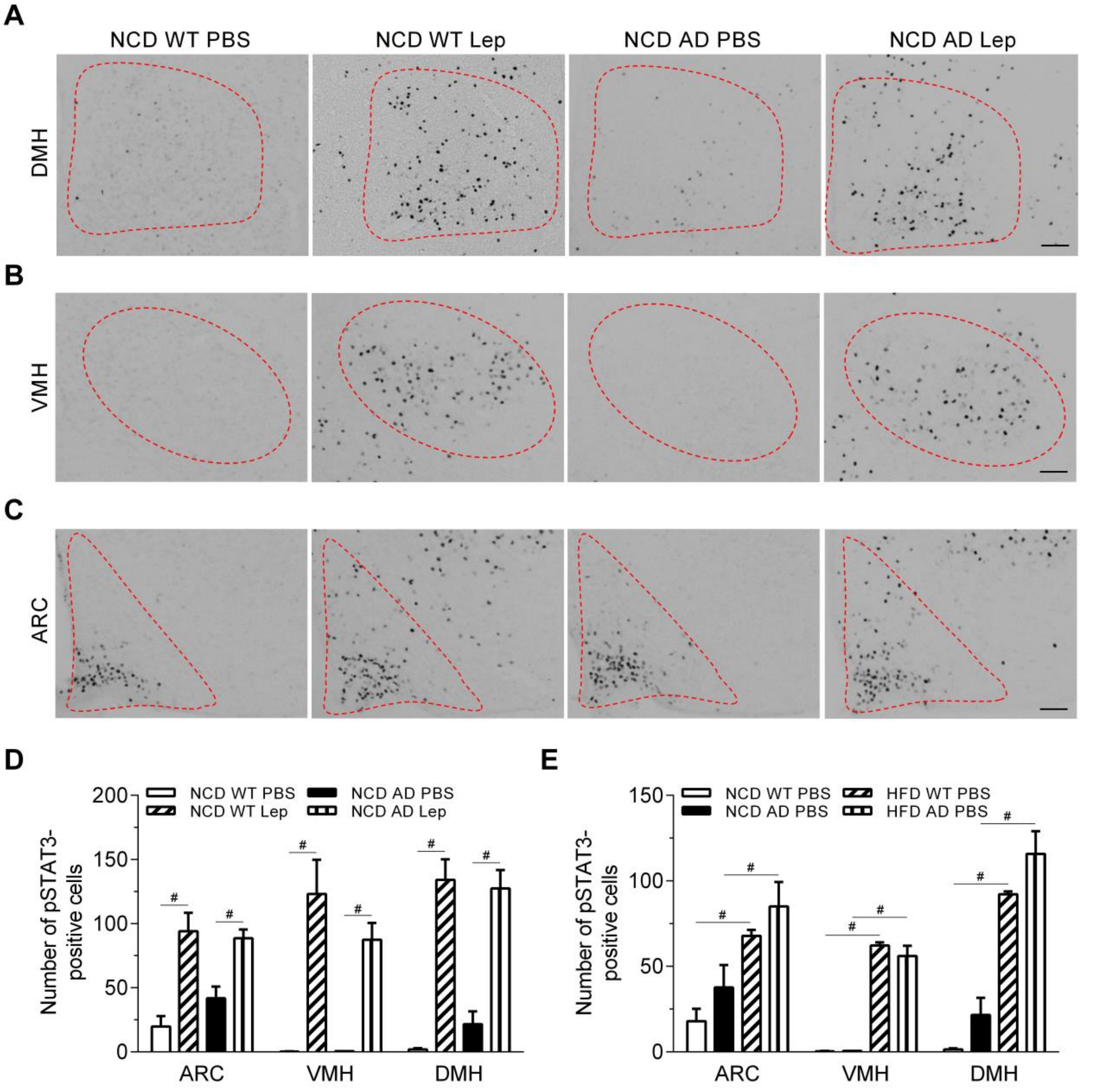

Supplementary Figure S3. HFD elevates the basal level of pSTAT3 in the mediobasal hypothalamus. (**A** – **C**) Representative immunohistochemical images of pSTAT3-positive cells in the ARC, VMH, and DMH of NCD WT and AD mice after the injection of leptin (Lep) or the vehicle (PBS). The third ventricle was at the left in each panel. The red dotted line defined the area of the DMH (**A**), VMH (**B**), and ARC (**C**) in which numbers of pSTAT3-positive cells was quantified. Scale bar, 55 μm (**A**) and 50 μm (**B,C**). (**D**) The numbers of pSTAT3-positive cells of NCD WT mice after the injection of leptin or PBS were quantified (n = 3-4/group). (**E**) The numbers of pSTAT3-positive cells of four groups injected with PBS were compared (n = 3-5/group). Data was expressed as mean ± SEM. At least three independent experiments were performed. Statistically different between groups were labeled with # ($p < 0.05$), determined by one-way ANOVA and Tukey's HSD post-hoc tests.

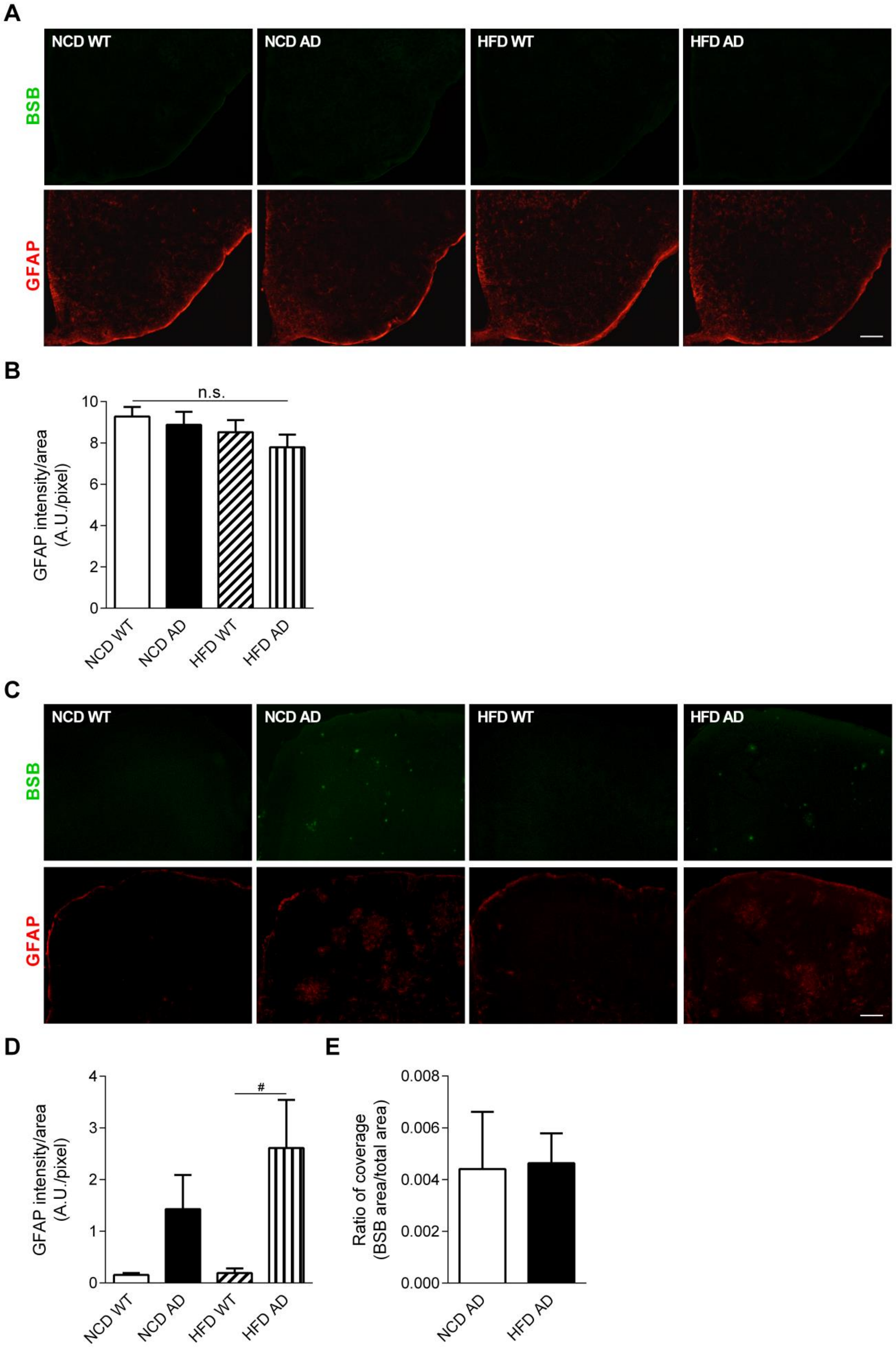

Supplementary Figure S4. Senile plaques and reactive astrocytes are present in the cortex, but not the hypothalamus of NCD and HFD AD mice. (**A**) Representative fluorescent images after BSB staining (green, upper panel) and GFAP immunoreactivity (red, lower panel) in the mediobasal

hypothalamus. The third ventricle was at the left in each panel. Scale bar, 50 μm. (**B**) The intensity of GFAP in the ARC of four groups was quantified (n = 4-6/group). (**C**) Representative fluorescent images of senile plaques stained by BSB (green, upper panel) and GFAP immunoreactivity (red, lower panel) in the cortex. Scale bar, 50 μm. (**D,E**) The intensity of GFAP and the area of senile plaques in the cortex were quantified (n = 4-5/group). Data was expressed as mean ± SEM. At least three independent experiments were performed. Statistically different between groups were labeled with # ($p < 0.05$), determined by one-way ANOVA and Tukey's HSD post-hoc tests (**B,D**).

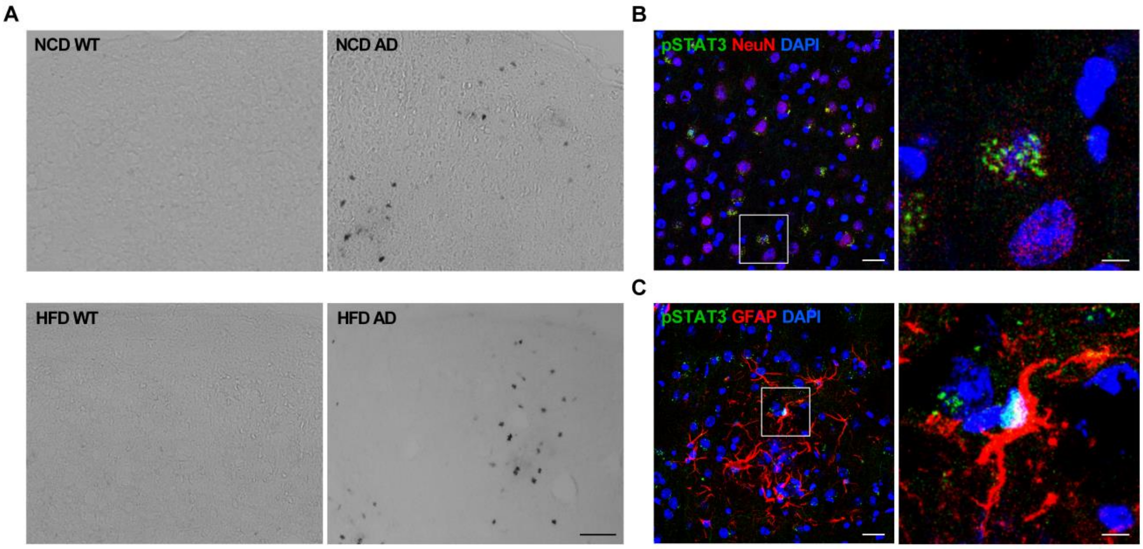

Supplementary Figure S5. Phosphorylated STAT3 in neurons and astrocytes of AD mice. (**A**) Representative images of pSTAT3-positive cells in the cortex of NCD and HFD AD mice. Scale bar, 50 μm. (**B**) Representative confocal images of pSTAT3 (green) and NeuN (red) immunoreactivity (upper left panel). Scale bar, 20 μm. Magnified images of box area (upper right panel). Scale bar, 5 μm. (**C**) Representative confocal images of pSTAT3 (green) and GFAP (red) immunoreactivity (lower left panel). Scale bar, 20 μm. Magnified images of box area (lower right panel). Scale bar, 5 μm. Nuclei were counterstained with DAPI.
